# Antidiabetic effects of the ethanolic extract of *Allium saralicum* R.M. Fritsch on streptozotocin‐induced diabetes in a mice model

**DOI:** 10.1002/fsn3.2405

**Published:** 2021-06-18

**Authors:** Simin Fazelipour, Mahsa Hadipour Jahromy, Zahra Tootian, Nader Goodarzi

**Affiliations:** ^1^ Department of Anatomy Faculty of Medicine Tehran Medical Sciences Islamic Azad University Tehran Iran; ^2^ Herbal Pharmacology Research Center Faculty of Medicine Tehran Medical Sciences Islamic Azad University Tehran Iran; ^3^ Department of Basic Sciences Faculty of Veterinary Medicine University of Tehran Tehran Iran; ^4^ Department of Basic Sciences and Pathobiology Faculty of Veterinary Medicine Razi University Kermanshah Iran

**Keywords:** *Allium saralicum*, anemia, diabetes, liver, mice, streptozotocin

## Abstract

Medicinal plants can protect different organs against diabetes‐induced oxidative stress due to their antioxidant compounds. The present study was designed to investigate the potential of *Allium saralicum* R.M. Fritsch (A. saralicum) ethanolic extract to alleviate the adverse effects of streptozotocin (STZ)‐induced diabetes in male BALB/c mice. Seventy male mice were randomly divided into seven groups (*n* = 10). Diabetes was experimentally induced by STZ (60 mg/kg bw). *A*. *saralicum* ethanolic extract with doses 5, 20, 80, and 320 mg/kg was administrated for 20 consecutive days in diabetic animals. Based on the obtained results, the untreated diabetic mice showed high blood glucose level, cholesterol, low‐density lipoprotein (LDL), white blood cells count (WBC), and platelets, as well as liver enzymes, urea, and creatinine. Administration of different doses of *A*. *saralicum* extract significantly reduced blood glucose level similar to glibenclamide. Also, the levels of catalase and superoxide dismutase enzymes restored toward normal level. All hepatic and renal function parameters as well as hematological parameters were improved following treatment with *A*. *saralicum* extract particularly at high doses. Histopathological studies showed a decrease in hepatic, renal, and pancreatic damage after treatment with *A*. *saralicum* extract. The results of the present work indicate that *A*. *saralicum* ethanolic extract can attenuate diabetic hepato‐renal, pancreatic, and hematological damages.

## Graphical abstract

Ethanolic extract of Allium saralicum R.M. Fritsch contains antioxidant compounds such as linolenic acid, phytol, and neofitadine. Ethanolic extract of Allium saralicum R.M. Fritsch can improve hyperglycemia caused by diabetes, and attenuated renal, hepatic, and pancreatic complications. Ethanolic extract of Allium saralicum R.M. Fritsch can prevent anemia and blood disorders following diabetes by controlling the hematological parameters.

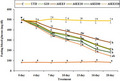

## 1 INTRODUCTION

Diabetes mellitus is a common metabolite disorder in both developing and developed countries (Kaczmarczyk‐Sedlak et al., 2019). In this regard, the body ability to yield insulin reduce or insulin resistance occurs in the body (Kayar & Agin, 2019). The main clinical complications of the diabetes mellitus are weak cutaneous wound healing, reduced fibrinolytic activity, severe chronic atherosclerosis, hypertension, dyslipidemia, disturbed hematological parameters including erythrocyte aggregation, erythrocyte deformability hematocrit and plasma proteins, retinal failure, and renal failure (Jabłońska et al., 2019; Szymusik et al., 2019).

Streptozotocin (STZ), which is used for inducing diabetes, is a very toxic agent for pancreas cells especially α and β cells (Brosius et al., 2009; Michalak et al., 2020). STZ causes DNA inconvenience and apoptosis in α and β cells as a nitrosourea class alkylating agent (Lenzen, 2008; Tesch & Allen, 2007). In addition to α and β cells, the liver and kidney are also sensitive to the toxicity of STZ (Rerup, 1970; Weiss, 1982), making it arduous to differentiate between diabetic hepatopathy and nephropathy (Tay et al., 2005).

Previous studies have indicated that STZ causes diabetes by changing the situation of antioxidant enzymes (Weiss, 1982). The results of many reports have revealed that ethnomedicinal plants by increasing the antioxidant enzymes levels have significant potentials for protecting of the pancreas, kidney, and liver against several toxins such as STZ (Hagh‐Nazari et al., 2017; Najafi et al., 2017). Some medicinal herbs have unique content of alkaloids, naphtha‐quinone, tannins, triterpenes, saponins, and flavonoids (Goodarzi et al., 2016).

Iran, as an old civilized country, has a long history of medical sciences with celebrities appreciated and acknowledged worldwide (Goodarzi et al., 2016; Goorani et al., 2018; Sherkatolabbasieh et al., 2017). Iranian traditional medicine is one of the main traditional medicines in the world. Previously, *Satureja khuzistanica*, *Opuntiastrepta cantha*, *Silybum marianum*, *Ginkgo biloba*, *Trigonella foenum*, *Ipomoea betatas*, *Citrullus colocynthis*, *Ocimum sanctum*, *Vaccinumarctos taphylos*, *Plantago ovate*, *Securigera Securidaca*, *Allium sativum*, *Cuminum cyminum,* and *Panax* *ginseng* have been examined to treat diabetes in Iranian and Asian medicines (Patti et al., 2017; Shojaii et al., 2011).

In this regard, it is predicated that *Allium saralicum* (*A*. *saralicum*) leaves have significant antidiabetic properties. The antioxidant compounds of *Allium* genus are Allicin [diallyl thiosulfinate], tuberoside M, thiosulfinates, S‐propargyl‐L‐cysteine, quercetin, S‐benzyl‐cysteine, diosgenin, polysulfanes, diosgenin, fisetin, diallylpolysulfides, onionin A, flavonoids, furostanol saponins, allyl sulfides, saponins, glycosides, diallyl tetrasulfide, and garlic oil (Goodarzi et al., 2016; Sherkatolabbasieh et al., 2017). Previously, the gastroprotective, anticancer, hepatoprotective, nephroprotective, anti‐inflammatory, antiobesity, antidiabetic, immunoprotective, antiparasitic, neuroprotective, antifungal, antiviral, and antibacterial effects of *A*. *saralicum* have been proven (Foroughi et al., 2016). Antidiabetic properties of the *Allium* genus can be related to the S‐benzyl‐cysteine, diosgenin, polysulfanes, diosgenin, fisetin, allicin[diallyl thiosulfinate], tuberoside M, S‐allylmercaptocysteine, thiosulfinates, S‐propargyl‐L‐cysteine, Ace‐AMP1, and quercetin (Patti et al., 2017). In Iranian medicine, this plant is used to treat several types of metabolite disorders such as diabetes (Foroughi et al., 2016).

Therefore, in the present experiment, we aimed to survey antidiabetic potentials of *A*. *saralicum* ethanolic extract in a mice model with focusing on biochemical, hematological, and histopathological approaches.

## 2 MATERIAL AND METHODS

### 2.1 Plant sample collection

In this research, *A*. *saralicum* was collected from the mountains of Kermanshah city (34.3277° N, 47.0778° E), Iran in May 2018. AS was identified by the Agriculture Faculty Research Center Herbarium, Razi University, Iran (No. 2738RUH).

### 2.2 Extract preparation

*A. saralicum* leaves were collected in summer, then milled after drying. 220 g of leaf powder was dissolved in 100% alcohol solution for 2 days. Then, the solution was filtered through paper (Whatman filter paper no.42, Millipore, USA. Cat No. 1442125) and dried in room temperature. Finally, 30 g of *A*. *saralicum* ethanolic extract was stored at 4℃ (Sherkatolabbasieh et al., 2017). The obtained extract was used for LC‐Mass analysis.

### 2.3 Animals

7‐week‐old male BALB/c mice (*n* = 70) were purchased from the Pastor Institute, Tehran, Iran. The mice were housed in 12 hr of light–dark, at 24–33℃, 45%–65% relative humidity and fed with standard pellet (Crude Protein: 23% Crude Fat: 3.0% Crude Fiber: 7.0% Acid Insoluble Ash 8% Calcium: 1%–2.5% Phosphorus: 0.9% Sodium: 0.5%–1% Moisture: 12%) and water ad libitum. The protocols were approved by the Ethics Committee of Tehran Medical Sciences, Islamic Azad University (Approval no: IR.IAU.PS.REC.1398.214) and performed completely in line with the guidelines of the Animal Ethics Committee.

### 2.4 Diabetes induction

Diabetes was induced by intraperitoneal injection of a single dose of STZ (Sigma, St. Louis, MO, USA) dissolved in citrate buffer (0.1 M and pH 4.5), at a dose of 60 mg/kg body weight. The animals with fasting blood glucose more than 350 mg/dl were considered as diabetic (Hagh‐Nazari et al., 2017).

### 2.5 Experimental design

One day after induction of diabetic, the animals were classified into the several groups (*n* = 10) and treated through gavage for 20 days:

1. C: Healthy group treated by 0.5 ml normal saline.
2. UTD: Diabetic group treated by 0.5 ml normal saline.
3. G20: Diabetic group treated by 0.5 ml glibenclamide (20 mg/kg).
4. ASEE5: Diabetic group treated by 0.5 ml *A*. *saralicum* ethanolic extract (ASEE) (5 mg/kg).
5. ASEE20: Diabetic group treated by 0.5 ml ASEE (20 mg/kg).
6. ASEE80: Diabetic group treated by 0.5 ml ASEE (80 mg/kg).
7. ASEE320: Diabetic group treated by 0.5 ml ASEE (320 mg/kg).

The dose selection was performed according to the previous studies (Sherkatolabbasieh et al., 2017).

### 2.6 Blood sampling

For measuring the fasting blood glucose, the blood was taken seven times (1–20 days) from the tail vein and asses by a glucometer. On day 20 of the experiment, 6 mg/kg of xylazine and 38 mg/kg of ketamine HCl were injected into the tail vein for euthanizing the animals. Then, the bloods were extracted from the hearts for biochemical and hematological experiments.

### 2.7 Determination of biochemical parameters

The collected samples were centrifuged for 16 min at 12,000 rpm and serum separated. In serum, the levels of alkaline phosphatase (ALP), alanine aminotransferase (ALT), aspartate aminotransferase (AST), albumin, total protein, conjugated bilirubin, total bilirubin, creatinine, urea, cholesterol, low‐density lipoprotein (LDL), and high‐density lipoprotein (HDL) were analyzed by using diagnostic kits in Mehr laboratory, Iran.

### 2.8 Determination of hematological parameters

In the hematological section, the blood samples were examined by a hematology analyzer. The parameters including white blood cell (WBC), red blood cell (RBC), hemoglobin (Hb), packed cell volume (PCV), mean corpuscular volume (MCV), mean corpuscular hemoglobin (MCH), and mean corpuscular hemoglobin concentration (MCHC) were assessed.

### 2.9 Evaluation of the endogenous antioxidant enzymes activities

In this research, the levels of liver and kidney antioxidant enzymes, that is, catalase (CAT) and superoxide dismutase (SOD) were measured according to the Mohammadi et al., (2020) and Hemmati et al., (2020) methods, respectively.

### 2.10 Histopathological assessment

In the histopathological section of the recent study, the pancreas, liver, and kidney samples were collected and investigated after preparing tissue sections. The volume density of the islets and B cells, percentage of B cells, number of islets, and average area of islets were measured. In the liver sections, the enlargement and congestion in sinusoids, central veins, portal veins, and hepatic arteries, sinusoids hyperemia, fibrin and mononuclear cells leakage in pericentral veins and periportal zones, bile ducts proliferation, hepatocytes cellular and nuclear pleomorphism, eosinophilic cytoplasmic bodies and inclusion bodies in hepatocytes, hepatocytes necrosis, and liver fibrosis and cirrhosis were evaluated.

In the kidney, the enlargement and congestion in glomeruli, renal veins, and renal arteries, fibrin leakage in periglomerular zone, perirenal veins, and perirenal arteries, proximal convoluted tubules and distal convoluted tubules, cells necrosis, glomerular and tubular atrophy, and renal fibrosis were assessed.

### 2.11 Statistical analysis

The normality of data was determined by Kolmogorov–Smirnov test and followed by one‐way ANOVA test and post hoc Duncan test. All of the statistical analyses were conducted using SPSS 22.0 (IBM SPSS Statistics for Windows, version XX) (IBM Corp.) and a *p* ≤ .05 was considered significant. The values are presented as mean ± *SD*.

## 3 RESULTS

### 3.1 Chemical composition of ASEE

The extract yield of *A*. *saralicum* was 6.25% (18.75g), calculated on the fresh leaves of the plant. Overall, fifteen compounds such as neophytadiene, 2‐hexadecene‐3,7,11,15‐tetramethyl, hexadecanoic acid, phytol, linolenic acid‐methyl ester, hexanedioic acid, bis (2‐ethylhexyl) ester, 1,4,8,11‐tetraazacyclotetradecane, hexatriacontane, nonadecene, ethanol‐2‐tetradecyloxy, γ‐tocopherol, eicosane, vitamin E, 2‐phenyl‐5‐methylindole, and n‐ethyl‐1,3‐dithioisoindoline were identified in the ASEE using GC‐MS, and linolenic acid‐methyl ester (24.39%), phytol (14.19%), and neophytadiene (11.6%) were the most detected compounds (Table 1) (24).

**TABLE 1:** The components of ASEE analyzed by GC/MS

No	Compound	Area (%)^a^	Retention time (min)
**1**	**Neophytadiene** ^b^	**11.6**	**18.319**
2	2‐Hexadecene, 3,7,11,15‐tetramethyl	1.4	22.133
3	Hexadecanoic acid	6.42	23.819
**4**	**Phytol** ^b^	**14.19**	**25.838**
**5**	**Linolenic acid, methyl ester** ^b^	**24.39**	**26.253**
6	Hexanedioic acid, bis(2‐ethylhexyl) ester	1.28	29.324
7	1,4,8,11‐Tetraazacyclotetradecane	1.28	30.072
8	Hexatriacontane	3.3	30.430
9	Nonadecene	5.67	32.572
10	Ethanol, 2‐tetradecyloxy	6	34.705
11	γ‐Tocopherol	3.03	36.926
12	Eicosane	2.89	37.502
13	Vitamin E	6.14	38.295
14	2‐Phenyl−5‐methylindole	6.82	42.228
15	N‐ethyl−1,3‐dithioisoindoline	2.16	44.283
**Total**		**96.57%**	

^a^ Expressed as percentage of the total peak area.

^b^ The dominant compounds are indicated in bold.

### 3.2 Effect of ASEE on fasting blood glucose concentration

**FIGURE 1:**
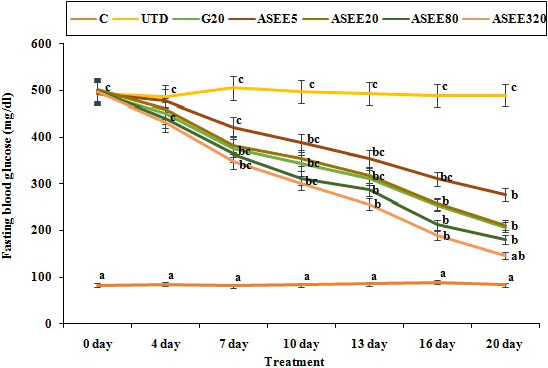
Fasting blood glucose levels on different days in the controls and ASEE‐treated groups

The effect of ASEE on fasting blood glucose level in the diabetic mice is presented in Figure 1. There was no significant change in the blood glucose level of the control mice throughout the study. The blood glucose levels of the untreated diabetic mice increased to approximately 520% (*p* ≤ .05) in a time‐dependent manner. However, treatment of the diabetic mice with ASEE at all doses significantly (*p* ≤ .05) decreased the blood glucose levels similar to the glibenclamide‐treated mice at day 20 of the experiment. ASEE exerted its maximum effect on day 20 of the experiment.

### 3.3 Histopathological findings

**FIGURE 2:**
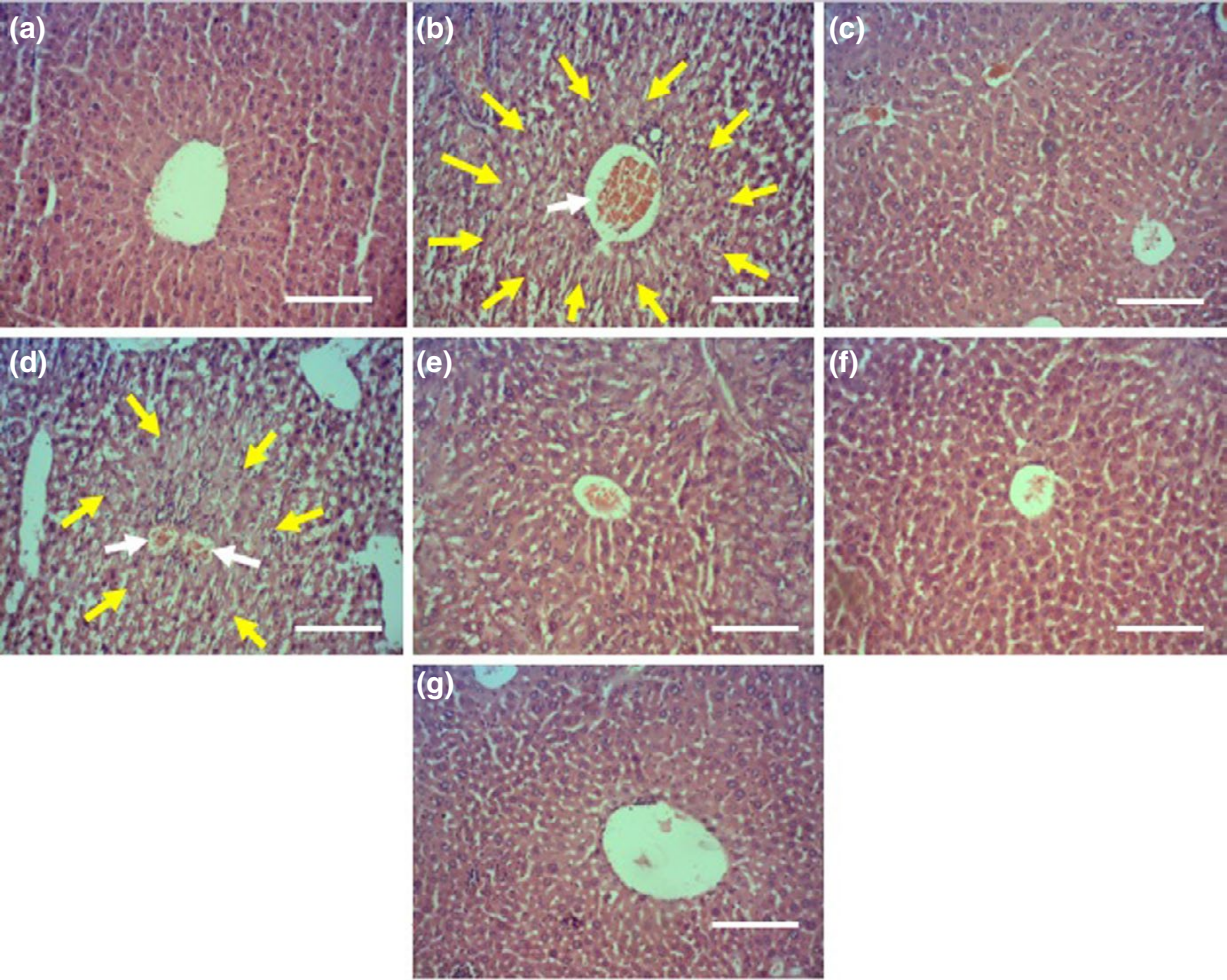
The effects of ASEE on histological structure of rat's liver. The histological appearance of liver in (a) Control, (b) non‐treated diabetic, (c) glibenclamide‐treated, (d) diabetic +ASEE 5 mg/kg, (e) diabetic +ASEE 20 mg/kg, (f) diabetic +ASEE 80, and (g) diabetic +ASEE 320 mg/kg treated groups. The sections show normal structure in control, glibenclamid‐treated and ASEE320 mg/kg treated rats and necrotic and fibrotic changes in non‐treated diabetic rats. Yellow arrows: necrotic zone, White arrows: vascular congestion (H&E, 100×)

The histological sections of the liver in the untreated diabetic mice showed degenerative changes in the hepatocytes represented by disorganization of the hepatic cords, hyperemia of sinusoids, enlargement and congestion of the central veins, portal veins, and hepatic arteries with mild hepatocellular necrosis, fibrin, and mononuclear cells leakage. The hepatocytes of the untreated diabetic mice revealed morphological changes such as pyknosis, karyorrhexis, chromatolysis, and cytoplasmic vacuolization. However, the liver of the ASEE‐treated diabetic mice indicated significant improvement compared to those of the untreated diabetic ones except the presence of a few mildly degenerated hepatocytes around the central veins and some cytoplasmic vacuoles. In addition, there was no evidence of hemorrhages, inflammatory cells infiltration, or parenchymal cell necrosis in the livers of the ASEE320‐treated diabetic mice. The liver of the control group had normal structure (Table 2, Figure 2).

**TABLE 2:** Histopathological analysis of the liver in controls and ASEE‐treated groups

No	Liver Changes	C	UTD	G20	ASEE5	ASEE20	ASEE80	ASEE320
1	Enlargement of sinusoids	−	+++	+	+	+	−	−
2	Enlargement of central veins	−	+++	+	++	++	+	−
3	Enlargement of portal veins	−	+++	+	++	+	−	−
4	Enlargement of hepatic arteries	−	+++	+	+	+	−	−
5	Congestion in central veins	−	++++	++	++	++	+	+
6	Congestion in portal veins	−	++++	++	++	++	+	−
7	Congestion in hepatic arteries	−	+++	++	++	++	−	−
8	Fibrin leakage in pericentral veins	−	++	−	+	+	−	−
9	Fibrin leakage in periportal zones	−	+++	+	++	+	+	−
10	Mononuclear cell leakage in periportal zones	−	++++	++	++	++	+	+
11	Necrosis of hepatocytes	−	++++	+	+	+	+	−

**FIGURE 3:**
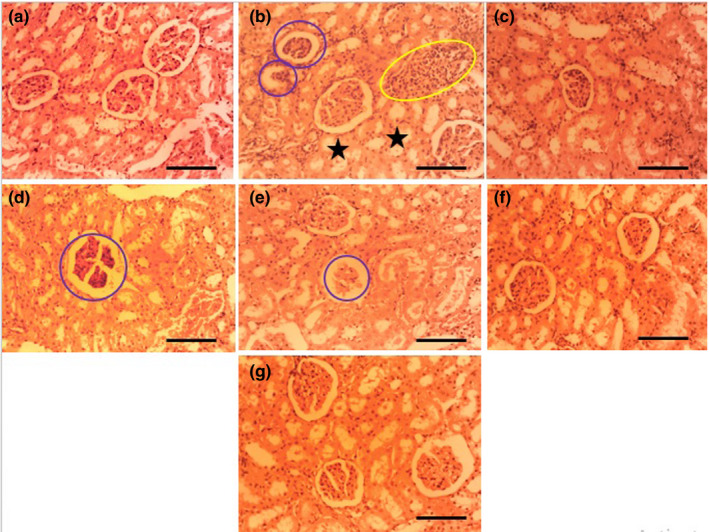
The effects of ASEE on histological structure of rat's kidney. The histological appearance of liber in (a) Control, (b) non‐treated diabetic, (c) glibenclamide‐treated, (d) diabetic +ASEE 5 mg/kg, (e) diabetic +ASEE 20 mg/kg, (f) diabetic +ASEE 80 and (g) diabetic +ASEE 320 mg/kg treated groups. The sections show normal structure in control, glibenclamid‐treated and ASEE320 mg/kg treated rats. Yellow circle: inflammatory cells infiltration, Blue circles: necrotic glomeruli, Black stars: necrotic tubules (H&E, 100×) (H&E, 100×)

The kidneys of the control and ASEE‐treated mice had normal structure. The proximal and distal convoluted tubules, renal corpuscles, glomerulus, and glomerular capsule had normal architecture, and in the untreated diabetic group, structural defects were seen in all of the above parameters. Microscopic examination of the kidneys of the diabetic mice treated with ASEE320 and ASEE80 did not show tubular necrosis or necrotic changes in the glomerular epithelium or glomerular and vascular hemorrhages (Table 3, Figure 3).

**TABLE 3:** Histopathological analysis of kidney in controls and ASEE‐treated groups

No	Kidney Changes	C	UTD	G20	ASEE5	ASEE20	ASEE80	ASEE320
1	Enlargement of glomeruli	−	++	−	−	−	−	−
2	Enlargement of renal vein	−	++	−	+	−	−	−
3	Enlargement of renal artery	−	++	−	−	−	−	−
4	Congestion in renal vein	−	+++	+	+	+	−	−
5	Congestion in renal artery	−	++	−	−	−	−	−
8	Fibrin leakage in perirenal vein	−	++	−	+	−	−	−
6	Fibrin leakage in perirenal artery	−	+	−	−	−	−	−
7	Necrosis of proximal convoluted tubule cells	−	++	−	+	+	−	−
8	Necrosis of distal convoluted tubule cells	−	++	−	−	−	−	−
9	Glomerular and tubular atrophy	−	+	−	−	−	−	−

**FIGURE 4:**
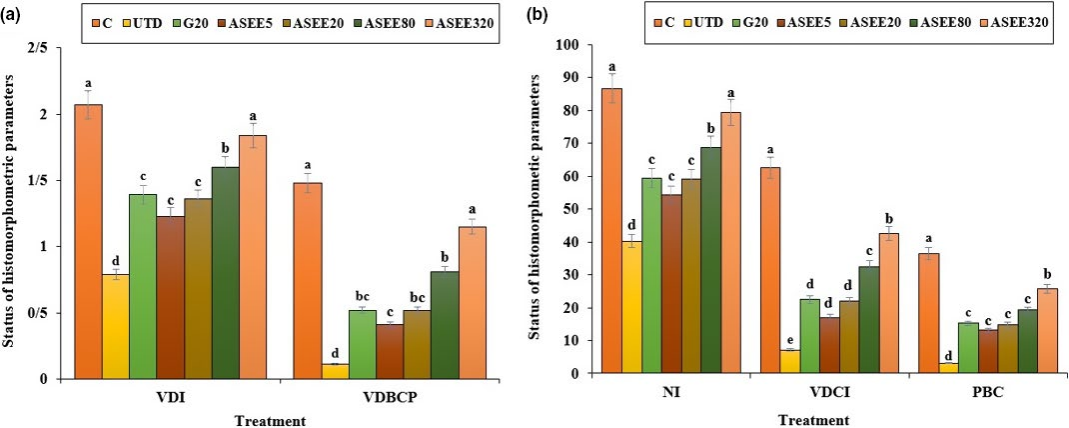
(a) VDI, and VDBCP and (b) NI, VDCI, and PBC values in the controls and ASEE‐treated groups. NI (Number of islets (per mm^2^)), VDCI (B cells volume density in islets), PBC (B cells percent), VDI (Islets volume density), and VDBCP (B cells volume density in pancreas)

The effect of ASEE on histomorphometric findings of the pancreatic tissue in the diabetic mice is presented in Figure 4. The number of pancreatic islets, volume density of the beta cells as well as the percentage of the beta cells showed a significant decrease (*p* ≤ .05) in the untreated diabetic mice compared to the normal control group. The volume density of pancreatic islets also showed a significant decline (*p* ≤ .05) following induction of diabetes. The pancreas of the diabetic mice treated with ASEE showed a slight increase in the size of pancreatic islets, having a few cells with hyperchromatic nucleus and regeneration of the beta cells in the center of islets. Also a regeneration process of pancreatic islets was more evident in ASEE‐treated groups. Although the number per square millimeter of the pancreatic islets, the volume density of the islets, and the volume density of the beta cells in the pancreas improved following administration of ASEE320, however, the percentage of beta cells, and the volume density of the beta cells in the pancreatic islets in the ASEE‐treated mice were still significantly (*p* ≤ .05) lower than those of the control group.

### 3.4 Effect of ASEE on liver biochemical parameters

**FIGURE 5:**
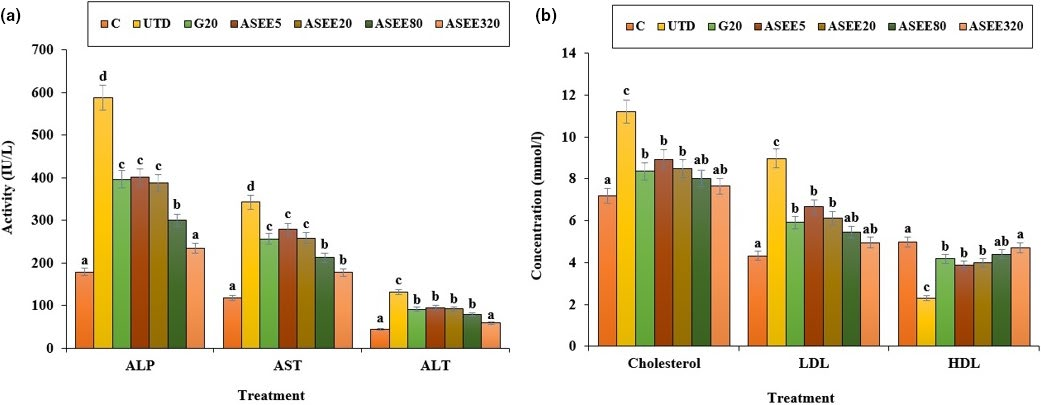
(a) The serum levels of ALP, AST, ALT (IU/L), and (b) cholesterol, LDL, and HDL (mmol/L) in the controls and ASEE‐treated groups

**FIGURE 6:**
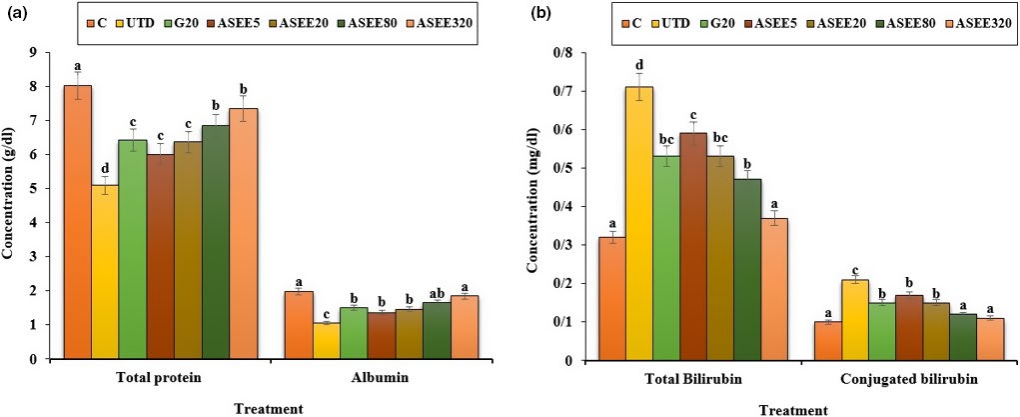
(a) Total protein and albumin levels and (b) Total bilirubin and conjugated bilirubin in the controls and ASEE‐treated groups

**FIGURE 7:**
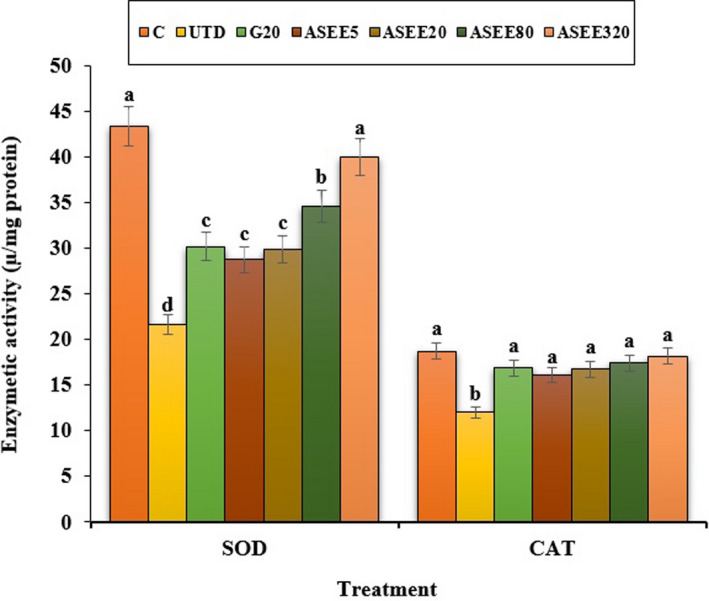
The serum levels of liver SOD and CAT in the controls and ASEE‐treated groups

The estimated values of the liver enzymes are presented in Figures 5, 6, 7. STZ‐induced hepatotoxicity increased ALP, AST, ALT, cholesterol, LDL, total, and conjugated bilirubin and decreased HDL, SOD, CAT, total protein, and albumin significantly (*p* ≤ .05) as compared to the control group. Several doses of ASEE and glibenclamide could significantly (*p* ≤ .05) decrease the raised levels of ALP, AST, ALT, cholesterol, LDL, total and conjugated bilirubin and increased HDL, SOD, CAT, total protein, and albumin significantly (*p* ≤ .05) as compared to the untreated group.

### 3.5 Effect of ASEE on kidney biochemical parameters

**FIGURE 8:**
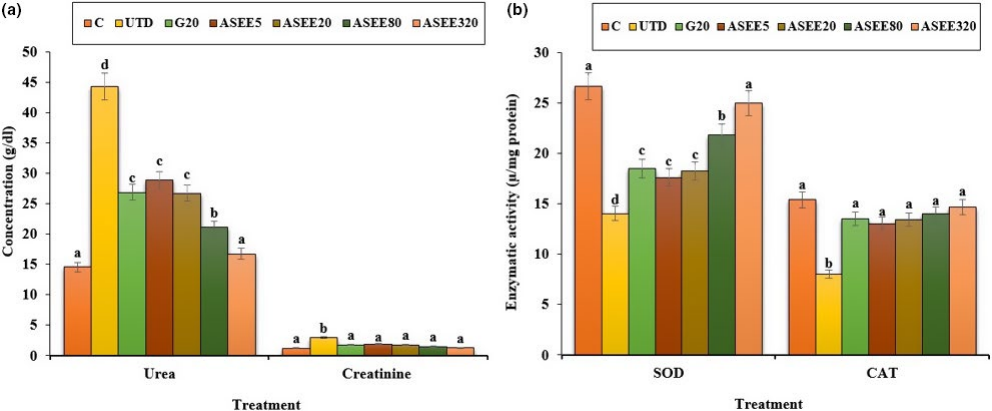
(a) The serum levels of urea, creatinine, and (b) kidney SOD and CAT levels in the controls and ASEE‐treated groups

The estimated values of the kidney biochemical parameters are depicted in Figure 8. STZ‐induced diabetes increased urea and creatinine levels and decreased CAT and SOD levels significantly (*p* ≤ .05) compared to the control group. Different doses of ASEE could significantly (*p* ≤ .05) ameliorate the above parameters. There was no significant difference in the above‐mentioned parameters (*p* ≤ .05) between ASEE320 and control groups.

### 3.6 Effect of ASEE on hematological parameters

**FIGURE 9:**
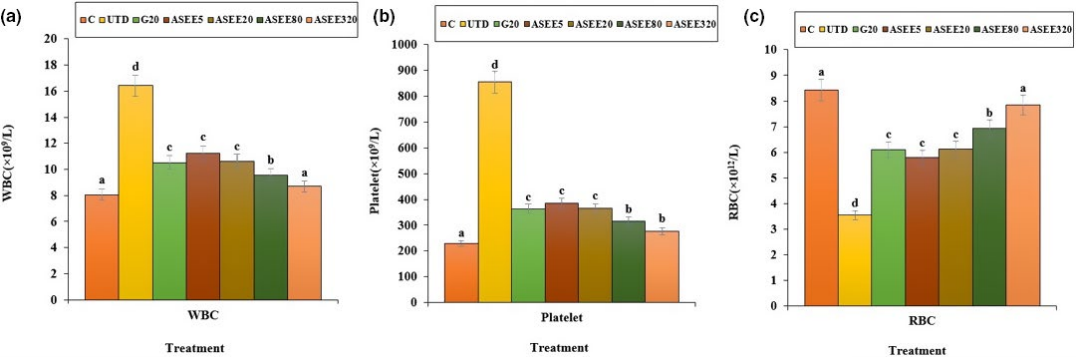
The number of (a) WBC, (b) platelet, and (c) RBC in the controls and ASEE‐treated groups

**FIGURE 10:**
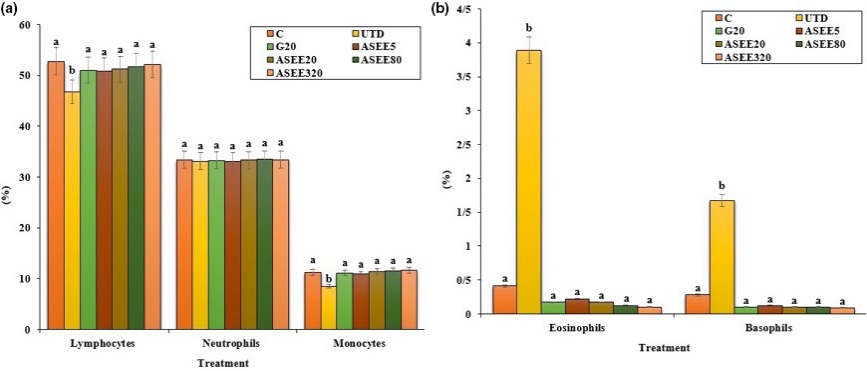
(a) Lymphocytes, neutrophils, monocytes, and (b) eosinophils and basophils percent in the controls and ASEE‐treated groups

**FIGURE 11:**
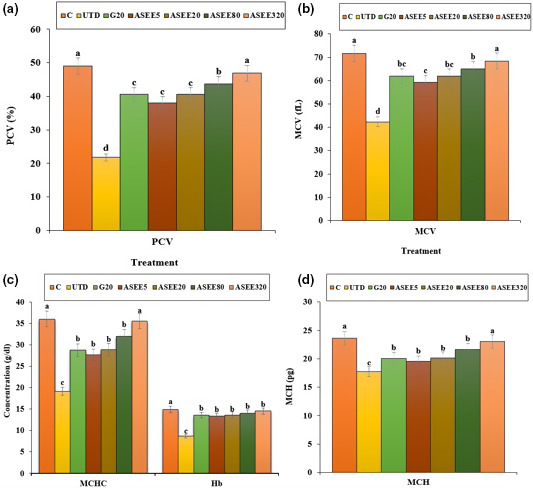
The values of (a) PCV, (b) MCV, (c) MCHC, and Hb and (d) MCH in the controls and ASEE‐treated groups

The number of WBC and platelet and percentage of eosinophils and basophils significantly (*p* ≤ .05) increased in the untreated diabetic mice. Also, the levels of lymphocytes, monocytes, RBC, MCV, Hb, MCH, MCHC, and PCV significantly (*p* ≤ .05) reduced in the untreated diabetic group. Treatment with ASEE significantly (*p* ≤ .05) ameliorated the above parameters. There were no significant differences in hematological parameters (*p* ≤ .05) between ASEE5, ASEE20, and glibenclamide groups. Also there were no significant differences (*p* ≤ .05) in the above factors (except for platelet and Hb levels) between ASEE320 and control groups (Figures 9, 10, 11).

## 4 DISCUSSION

The present study investigates the efficacy of ethanolic extract of *Allium saralicum* (*A*. *saralicum*) on streptozotocin‐induced diabetes in male mice from various histopathological, hematological, and biochemical aspects.

The obtained results showed that the ethanolic extract of *A*. *saralicum* could significantly reduce the blood glucose level in STZ‐induced diabetes. Such hypoglycemic effects of medicinal plants can be attributed to decrease in the rate and amount of intestinal absorption or increase in glucose uptake by peripheral tissues (Gupta et al., 2012; Hamden et al., 2001; Porchezhian et al., 2000). In addition, herbal extracts may have stimulatory effects on the remaining beta cells and more insulin production. Various studies have shown that the administration of plant extracts in laboratory diabetic animals can be effective in the reconstruction and repairment of the beta cells and Langerhans islands. Beta cells also have a remarkable potential for self‐renewing in the early stages of diabetes (Cumaoğlu et al., 2011; Pepato et al., 2004). Therefore, by default, it can be assumed that the ethanolic extract of *A*. *saralicum* is responsible for the production and secretion of insulin from recombinant beta cells in the pancreas. Based on the histopathological findings in this study, the volume density of the beta cells and also the number and size of the Langerhans islands showed a significant improvement in the diabetic mice treated with ethanolic extract of *A*. *saralicum*. These changes can explain declined blood glucose levels in these groups. In addition, many researchers have suggested that the antidiabetic effects of some of the natural extracts can be attributed to their insulin‐like effects, which enable them to decrease the blood glucose levels and serum lipids by controlling insulin (Shen et al., 2000; Zangeneh et al., 2018).

Dyslipidemia is one of the complications of hyperglycemia (Adeneye et al., 2010). This work showed that cholesterol and LDL levels decreased and HDL levels enhanced in the diabetic mice treated with ethanolic extract of *A*. *saralicum*. These results suggest that the ethanolic extract of *A*. *saralicum* has been able to improve the defect metabolism of the fatty acids in the streptozotocin‐induced diabetic mice. Increased lipid decomposition and the release of free fatty acids from peripheral tissues are other mechanisms for increasing the lipids profile in diabetes (Chaiyasut et al., 2011). Previous studies have shown that some compounds, especially saponins and steroids, exert antihyperlipidemic effects by preventing intestinal absorption of lipids and also by preventing the activity of lipase enzymes (Hamden et al., 2001). The increase in the liver enzymes in diabetic mice may be due to diabetes‐induced hepatic injuries (Rodrigues et al., 2010). The findings of the current work confirmed the signs of liver damage, such as dilation and congestion of the sinusoids, hepatic arteries, and veins. Treatment with different doses of *A*. *saralicum* ethanolic extract reduced the serum levels of transaminases and also improved the histopathologic alterations in the liver of the streptozotocin‐induced diabetic animals. Also, diabetes can increase the bilirubin levels directly by damaging the bile ducts or by releasing from the muscles (Gaamoussi et al., 2010). The obtained results showed that the conjugated and total bilirubin levels restored toward the normal levels after treating with high doses of ethanolic extract of *A*. *saralicum*.

It has been well established that stress oxidative plays a pivotal role in the pathogenesis of diabetes and vascular complications. Streptozotocin can increases reactive oxygen species (ROS) production and damages to the pancreas, leading to increased blood glucose level. These molecules are exacerbating factors in cellular injury, inflammation, cardiovascular diseases, and aging process. Therefore, antioxidants play a significant role in reducing diabetes complications (Rahimi et al., 2005; Tchinda et al., 2008). Based on the results of gas chromatography/spectrometry, linolenic acid is the most effective ingredient found in *A*. *saralicum* extract. According to the previous studies, this fatty acid known as herbal omega‐3 has antioxidant and anti‐inflammatory potentials and was used for treating various diseases such as experimental colitis, vascular thrombosis, osteoporosis, and myocardial infarction. This fatty acid has an excellent inhibitory effect on NO and iNOS production. Also, the antioxidant effects of linolenic acid can be attributed to its ability to regulate the expression of TNF‐α as well as inflammatory interleukins (Sherkatolabbasieh et al., 2017). Moreover, other compounds in *A*. *saralicum* extract, such as phytol, neofitadine, and vitamin E, are potent antioxidant and anti‐inflammatory agents (Ren & Chung, 2007). Hematological indices were another parameter studied in this study. In general, the relationship between diabetes and anemia has been fully documented in previous studies (Mehdi & Toto, 2009; Weiss & Goodnough, 2005). Several mechanisms can be considered for anemia associated with diabetes. Ferraro et al., (2011) in a study showed that diabetes affects the bone marrow cells and changes the microanatomy and physiology of the bone marrow stem cells. In addition, it seems that one of the causes of diabetes mellitus‐induced anemia is the glycosylation of the plasma membrane of the red blood cells. So that, hyperglycemia and protein oxidation lead to increased lipid peroxidation and ultimately diminish the fluidity and flexibility of the cell membrane and hemolysis of the red blood cells can be occurred (Kumar, 2012; Turk et al., 2002; Watala & Winocour, 1992). In this study, the number of white blood cells and platelets increased in the untreated diabetic animals, and the number of red blood cells, hemoglobin, MCV, MCH, MCHC, and PCV decreased significantly. Stookey et al., (2007) have shown that streptozotocin reduces the synthesis of MCH and MCHC, which indicates a defect in hemoglobin synthesis and a defect in osmotic pressure control and osmolality of the plasma. Treatment with *A*. *saralicum* extract, especially at high dose (320 mg/kg), improved the above‐mentioned parameters. Peelman et al., (2004) suggested that leptin and its receptor are responsible for hemopoiesis. Ohshita et al., (2004) showed that white blood cell count is associated with some diseases, including insulin resistance and diabetes (Pertynska‐Marczewska et al., 2004; Shurtz‐Swirski et al., 2001). The findings indicated that the *A*. *saralicum* ethanolic extract, in addition to improving the reduction of red blood cell count and related parameters, also increased leukocyte indices. Since oxidative stress is the main risk factor of hemolysis of the red blood cells and anemia due to diabetes, the improvement of hematological indices can be attributed to the antioxidant properties of *A*. *saralicum* extract on controlling catalase and superoxide dismutase enzymes.

## 5 CONCLUSION

In conclusion, according to the present results, it seems that the ethanolic extract of *A*. *saralicum* due to its antioxidant compounds such as linolenic acid, phytol, and neofitadine can improve hyperglycemia caused by diabetes, and attenuate renal, hepatic, and pancreatic complications. Also, this extract can prevent of anemia and blood disorders following diabetes by controlling the hematological parameters. These findings can be served as a light to formulate a suitable food supplement for alleviating diabetes complications in future.

## CONFLICT OF INTEREST

The authors declare that they do not have any conflict of interest.

## AUTHOR CONTRIBUTIONS

**Simin Fazelipour:** Conceptualization (equal). **Mahsa Hadipour Jahromi:** Investigation (equal). **Zahra Tootian:** Writing‐original draft (equal). **Nader Goodarzi:** Software (equal); Visualization (equal).

## ETHICAL APPROVAL

This study was approved by the Institutional Review Board of Tehran Medical Sciences, Islamic Azad University (Approval no: IR.IAU.PS.REC.1398.214).

## Data Availability

The data that support the findings of this study are available on request from the corresponding author. The data are not publicly available due to privacy or ethical restrictions.
